# A comparison of the test-negative and the traditional case-control study designs for estimation of influenza vaccine effectiveness under nonrandom vaccination

**DOI:** 10.1186/s12879-017-2838-2

**Published:** 2017-12-08

**Authors:** Meng Shi, Qian An, Kylie E. C. Ainslie, Michael Haber, Walter A. Orenstein

**Affiliations:** 1 Department of Biostatistics and Bioinformatics, Rollins School of Public Health, Emory University, Atlanta, 30322 USA; 2 Division of Infectious Diseases, Department of Medicine, School of Medicine, Emory University, Atlanta, 30322 USA

**Keywords:** Case-control study, Test-negative study, Probability model, Symptomatic influenza, Medically-attended influenza

## Abstract

**Background:**

As annual influenza vaccination is recommended for all U.S. persons aged 6 months or older, it is unethical to conduct randomized clinical trials to estimate influenza vaccine effectiveness (VE). Observational studies are being increasingly used to estimate VE. We developed a probability model for comparing the bias and the precision of VE estimates from two case-control designs: the traditional case-control (TCC) design and the test-negative (TN) design. In both study designs, acute respiratory illness (ARI) patients seeking medical care testing positive for influenza infection are considered cases. In the TN design, ARI patients seeking medical care who test negative serve as controls, while in the TCC design, controls are randomly selected individuals from the community who did not contract an ARI.

**Methods:**

Our model assigns each study participant a covariate corresponding to the person’s health status. The probabilities of vaccination and of contracting influenza and non-influenza ARI depend on health status. Hence, our model allows non-random vaccination and confounding. In addition, the probability of seeking care for ARI may depend on vaccination and health status. We consider two outcomes of interest: symptomatic influenza (SI) and medically-attended influenza (MAI).

**Results:**

If vaccination does not affect the probability of non-influenza ARI, then VE estimates from TN studies usually have smaller bias than estimates from TCC studies. We also found that if vaccinated influenza ARI patients are less likely to seek medical care than unvaccinated patients because the vaccine reduces symptoms’ severity, then estimates of VE from both types of studies may be severely biased when the outcome of interest is SI. The bias is not present when the outcome of interest is MAI.

**Conclusions:**

The TN design produces valid estimates of VE if (a) vaccination does not affect the probabilities of non-influenza ARI and of seeking care against influenza ARI, and (b) the confounding effects resulting from non-random vaccination are similar for influenza and non-influenza ARI. Since the bias of VE estimates depends on the outcome against which the vaccine is supposed to protect, it is important to specify the outcome of interest when evaluating the bias.

## Background

Influenza vaccine effectiveness (VE) has to be re-estimated in every season because predominant influenza virus types, subtypes and phenotypes change from one season to the next, necessitating a new vaccine targeting different strains in most seasons. As annual influenza vaccination is now widely recommended, randomized clinical trials for estimating VE are no longer ethical in many populations, and observational studies based on patients seeking medical care for acute respiratory illnesses (ARI) are the most efficient, and hence most widely used option. However, observational studies for estimating VE are prone to multiple sources of bias.

In this paper we present a new probability model for comparing the bias and precision of VE estimates from two popular case-control study designs under nonrandom vaccination, i.e., vaccination probabilities may depend on a covariate. In both study designs, ARI patients seeking medical care who test positive for influenza infection are considered cases. In the test-negative (TN) design, ARI patients seeking medical care who test negative for influenza infection serve as controls, while in the traditional case-control (TCC) design, controls are randomly selected individuals who did not contract an ARI, usually from the same community from which the cases came. The TN design was introduced in 2007 [1], and most of the influenza VE case-control studies conducted since then have used this study design. However, TCC studies are still being used occasionally [2–4]. TCC studies are usually costlier and more resource intensive due to the need to recruit controls through a separate mechanism.

Estimates of VE from case-control studies may be subject to the following sources of bias:

**(a) Probabilities of non-influenza ARI may depend on vaccination status:** In TN studies, individuals with non-influenza ARI serve as controls. Therefore, TN studies may produce biased estimates of VE unless vaccinees and non-vaccinees are equally likely to develop non-influenza ARI. The validity of this assumption has not yet been confirmed. De Serres et al. [5] used data from randomized clinical trials to argue that this assumption is usually satisfied. However, a randomized influenza vaccine trial [6] found that vaccinees had a significantly increased risk of virologically-confirmed non-influenza infection (that may lead to ARI) as compared to those who received the placebo.

**(b) Probabilities of influenza and non-influenza ARIs may depend on confounders:** Covariates such as health status, age, exposure, education and socio-economic status may be associated with both the likelihood of being vaccinated and the likelihood of developing influenza and non-influenza ARIs.

**(c) Vaccination may affect probability of seeking medical care in influenza patients:** Several studies [7–9] suggest that vaccinated individuals who contract influenza may have milder symptoms than unvaccinated influenza patients, and therefore may be less likely to seek medical care. This effect of vaccination is not expected to change health-care-seeking behavior of non-influenza ARI patients.

**(d) Probabilities of seeking medical care against ARIs may depend on confounders:** Since only ARI patients who seek medical care may be included in a TNC study, and only influenza patients who seek care may be included as cases in TCC studies, covariates that are associated with both the likelihood of being vaccinated and the likelihood of seeking care against ARI may contribute to the bias of influenza VE estimates.

**(e) Misclassification bias:** Diagnostic tests for influenza viruses are not 100% sensitive and specific. Vaccination status may also be misclassified.

In this work we consider the first four sources of bias. To focus on these sources, we ignore misclassification biases which are known to result in negative bias (i.e., bias toward lower estimation of VE) and are common to all studies that rely on results of diagnostic tests.

The goal of this article is to evaluate and compare the bias and precision of estimates of VE resulting from TN and TCC studies. As we will see, the bias of VE estimates may depend on the outcome of interest, i.e., the outcome against which the vaccine is expected to protect. We consider two outcomes of interest, symptomatic influenza (SI) and medically-attended influenza (MAI). In both the TN and TCC study designs, only influenza patients seeking medical care are considered cases. Therefore, one expects these study designs to produce estimates of VE against MAI. However, the media usually reports VE estimates from these case-control studies as ‘vaccine effectiveness against influenza’, without including the ‘medically-attended’ clause. As a result, the public may interpret these estimates as the effectiveness of the vaccine against *any influenza illness*, i.e., VE against SI. One of the objectives of this work is to highlight the importance of (a) clearly specifying the outcome against which the vaccine is supposed to protect, and (b) understanding that the bias of a VE estimate may be different for the two outcomes of interest.

We will (a) evaluate the bias of each of the VE estimates for each of the outcomes by comparing the expected value of the estimate with the true VE, and (b) evaluate the standard errors of the VE estimates. To perform these evaluations and comparisons, we developed a detailed stepwise probability model of the process involved in collecting data in these studies and deriving VE estimates. The model includes a covariate, health status, that may be associated with both the likelihood of being vaccinated and the propensity of seeking medical care against ARI. This allows us to assess the effects of nonrandom vaccination on the bias of VE estimates.

## Methods

We first describe the real-life process involved in conducting the two types of case-control studies and obtaining the estimates of VE. We then describe the model we developed to mimic this process.

### The study population

The source population for both types of case-control studies consists of all individuals receiving most of their medical care at a single clinic or at a specific network of clinics. Since influenza VE varies by age, we can assume that the model pertains to a subpopulation corresponding to a single age group.

### The study designs

When a member of the study population develops an ARI, s/he may decide to report to a clinic for treatment. At the clinic, the health care provider may ask the person to be tested for influenza viruses. If the person agrees then a swab is taken and sent to a laboratory for testing. In both study designs, a person who tests positive is eligible to be considered a case. In a TN study, an individual who tests negative is eligible to be considered a control. In a TCC study, controls are randomly selected members of the study population who have not developed ARI prior to their inclusion in the study. Usually, one or more controls are selected right after a case is identified. In both study designs, the vaccination status of every case or control is determined from manual or electronic records, or from oral histories.

### Outcome of interest and true VE

In this work we evaluate estimates of VE when the outcome of interest is either SI or MAI. SI is sometimes called ‘influenza illness’ or ‘influenza ARI’. Surveillance for SI is needed in the entire study population, and for persons ill with compatible illnesses, samples of influenza are taken for verification. A person is considered a true case of SI if s/he has an ARI and is infected by an influenza virus. For MAI, a true case is defined as a person who is influenza-infected, develops an ARI, and seeks medical care. In both cases, the true VE is defined as one minus the ratio of the probability of the outcome of interest in vaccinees and non-vaccinees.

### Estimation of VE and bias of VE estimates

In this work we focus on identifying the main sources of bias and their effects on the performance of the VE estimates. Some of these biases can be adjusted for in the analysis, but this is beyond the scope of the current work. In case-control studies, VE is usually estimated as one minus the odds ratio (OR) of being vaccinated in cases vs. controls. The bias of the estimate is defined as the difference between the expectation of the estimated VE and the true VE.

### The model

The model we developed for comparing the estimates from the two study designs follows the scheme described above with a few simplifications. We assume that (a) when a person seeks medical care for ARI then her/his probability of being tested for influenza viruses does not depend on vaccination status, health status, or on the actual cause of ARI (influenza/non-influenza); (b) given a person’s symptoms and influenza infection status, the sensitivity and specificity of the test do not depend on the tested person’s vaccination or health status; (c) a person’s vaccination status is determined without error; and (d) controls in a TCC study are selected at random from all asymptomatic individuals in the study population (See “The study population” section).

Our model includes a covariate, health status, and we assume that a person’s probabilities of being vaccinated, developing an ARI, and seeking medical care against ARI may be associated with her/his health status. In this way, the model generates possible confounding effects linking vaccination status with the probabilities of being included in the study and of becoming a case or a control.

The model consists of five steps, where the value of a single variable is determined at each step. The probability distribution of this variable may depend on the values of the variables from the previous steps. Below we define the five steps, the associated variables, and the probabilities determining each variable’s distribution.

#### Step 1: Health status

A person can be classified as “healthy” or “frail”. Define a binary variable X, where *X*=1 for a “healthy” person and *X*=0 for a “frail” person. Denote *π*=*P*(*X*=1).

#### Step 2: Vaccination

A person may be vaccinated against influenza. Define a binary variable *V*, where *V*=1 for a vaccinated person. The probability of being vaccinated may depend on health status; therefore, denote *α*_*x*_=*P*(*V*=1|*X*=*x*), *x*=0,1.

#### Step 3: Influenza infection and ARI

During the influenza season, a person may become infected with an influenza virus and develop an ARI. This outcome is referred to as “influenza ARI” (FARI), where “F” stands for flu. A person may also develop an ARI not resulting from influenza infection. This outcome is referred to as “non-influenza ARI” (NFARI). We therefore define an outcome variable *Y* with 3 categories as follows: *Y*=0 for no ARI, *Y*=1 for NFARI, and *Y*=2 for FARI. The distribution of *Y* depends on the person’s vaccination status, *V*, and health status, *X*. We denote *β*_*vx*_=*P*(*Y*=1|*V*=*v*,*X*=*x*), *v*=0,1, *x*=0,1 and *γ*_*vx*_=*P*(*Y*=2|*V*=*v*,*X*=*x*) for *v*=0,1, *x*=0,1 with *β*_*vx*_+*γ*_*vx*_≤1 for all *v*,*x*. Here we assume the “leaky vaccine” model, in which the vaccine provides a reduction in the probability of influenza transmission to the vaccinated person, rather than complete immunity [10]. Under this model, a vaccinee has a lower probability of becoming infected than a non-vaccinee, but is not rendered completely immune from influenza infection.

#### Step 4: Seeking medical care for ARI

A person with ARI may seek medical care and, in this case, be tested for influenza viruses. We define a binary variable *M* with *M*=1 for a person seeking medical care for her/his ARI. The probability of this event depends on Y (only individuals with ARI seek medical care), and it may be different for FARI and NFARI patients. In addition, the conditional distribution of *M* given *Y* may depend on *X* and *V*. We therefore define *δ*_*yvx*_=*P*(*M*=1|*Y*=*y*,*V*=*v*,*X*=*x*), where *y*=1,2, *v*=0,1 and *x*=0,1.

In order to reduce the number of parameters, we make two simplifying assumptions regarding the probabilities of seeking medical care: (1) the effect of health status on probability of seeking medical care does not depend on vaccination status or type of ARI; (2) the effect of vaccination status on probability of seeking medical care does not depend on health status (but it may depend on type of ARI).

Define a “standard person” as a person with X = 0 and V = 0. For a “standard person”, we define *δ*_*SN*_, *δ*_*SF*_ as follows:

- *δ*_*SN*_=*P*(*M*=1|*Y*=1,*V*=0,*X*=0)=*δ*_100_
- *δ*_*SF*_=*P*(*M*=1|*Y*=2,*V*=0,*X*=0)=*δ*_200_

In addition, we define two multipliers:

- *λ* = multiplier for x = 1; *λ* does not depend on V and Y.
- *Ψ*_*F*_ = multiplier for v=1 only when y=2; *Ψ*_*F*_ does not depend on X.

*λ* is the ratio of the probabilities of seeking medical care comparing a healthy and a frail person. *Ψ*_*F*_ is the ratio of the probabilities of seeking care comparing a vaccinated and unvaccinated influenza ARI patient.

Then, { *δ*_*yvx*_} can be written in terms of *δ*_*SN*_, *δ*_*SF*_ and the multipliers *λ*, *Ψ*_*F*_ as follows:

- *δ*_100_=*δ*_*SN*_, *δ*_101_=*δ*_*SN*_∗*λ*, *δ*_110_=*δ*_*SN*_, *δ*_111_=*δ*_*SN*_∗*λ*.
- *δ*_200_=*δ*_*SF*_, *δ*_201_=*δ*_*SF*_∗*λ*, *δ*_210_=*δ*_*SF*_∗*Ψ*_*F*_, *δ*_211_=*δ*_*SF*_∗*λ*∗*Ψ*_*F*_.

Note: The multiplier *Ψ*_*F*_ reflects the effect of severity of ARI in an influenza infected person. We assume that vaccination may reduce severity of symptoms, hence a vaccinated influenza patient may be less likely to seek care than an unvaccinated patient.

#### Step 5: Testing for influenza infection.

Although only individuals who seek medical care for an ARI are tested for influenza infection, it will be convenient to define a binary variable *T* as the (possibly unobserved) test result for any person with an ARI, regardless of whether or not s/he is actually tested. Define *T*=1 (*T*=0) if a person would test positive (negative) for influenza if tested. Because of assumption (b) above, the probability of testing positive given the person’s influenza infection status does not depend on *X*, *V*, or *M*. Denote *τ*_*y*_=*P*(*T*=1|*Y*=*y*) for *y*=1,2. Note that *τ*_1_ is one minus the test’s specificity and *τ*_2_ is the test’s sensitivity. In this study, we assume the test has 100% sensitivity and 100% specificity, i.e. *P*(*T*=1|*Y*=1)=*τ*_1_=0 and *P*(*T*=1|*Y*=2)=*τ*_2_=1.

**Fig. 1:**
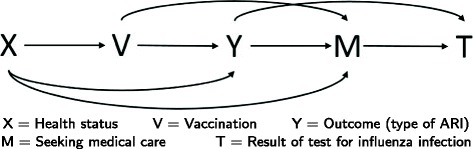
DAG of the model

Figure 1 shows the directed acyclic graph (DAG) of the model. Recent papers by Sullivan et al. [11] and Lipsitch et al. [12] discuss the use of DAGs to explore sources of bias of VE estimates from TN studies. A summary of the variables and parameters in our model is given in Table 1.

**Table 1:** List of parameters and other notation

Symbol	Definition	Values
X	Health status	0 - frail
		1 - healthy
V	Vaccination status	0 - unvaccinated
		1 - vaccinated
Y	ARI and influenza infection status	0 - no ARI
		1 - NFARI
		2 - FARI
M	Seeking medical care for ARI	0 - no
		1 - yes
T	Result of test for influenza infection	0 - negative
		1 - positive
*C*_*A*_	Case/control status in TND study	0 - control
		1 - case
*C*_*B*_	Case/control status in TCC study	0 - control
		1 - case
B	Participating in TCC study	0 - no
		1 - yes
*π*	Probability of having better health status (i.e. healthy persons)	0.7
*α*_*x*_	Probability of being vaccinated for a person of health status x	
*β*_*vx*_	Probability of NFARI for a person of vaccination status *v* and health status *x*	
$\rho _{\beta } = {{\beta _{1x}}\over {\beta _{0x}}}$	Ratio comparing vaccinees and non-vaccinees w.r.t. probability of NFARI	0.5-2.0
$\eta _{\beta } = {{\beta _{v1}}\over {\beta _{vo}}}$	Ratio comparing healthy and frail persons w.r.t. probability of NFARI	0.5-1.0
*γ*_*vx*_	Probability of FARI for a person of vaccination status *v* and health status *x*	
$\rho _{\gamma } = {{\gamma _{1x}}\over {\gamma _{0x}}}$	Ratio comparing vaccinees and non-vaccinees w.r.t. probability of FARI	0.4^a^
$\eta _{\gamma } = {{\gamma _{v1}}\over {\gamma _{v0}}}$	Ratio comparing healthy and frail persons w.r.t. probability of FARI	0.5-1.0
*δ*_*SN*_	Probability of seeking medical care for ARI for an unvaccinated frail person with NFARI	
*δ*_*SF*_	Probability of seeking medical care for ARI for an unvaccinated frail person with FARI	
*λ*	multiplier for the probability of seeking medical care for FARI or NFARI for a healthy person	0.5-2.0
*Ψ*_*F*_	multiplier for the probability of seeking medical care for FARI for a vaccinated person	0.5-1.0
*τ*_*e*_	Probability that a person of illness/infection status *e* tests positive for influenza infection	*τ*_1_=0, *τ*_2_=1

^a^Assumes a true VE of 60%. Thus, the probability of FARI in a vaccinee is 40% that of a non-vaccinee

### True VE in our model

When we evaluate the true VE, we assume that vaccination is done *at random*, i.e. for true VE we assume that vaccination status does not depend on health status *X* (*α*_0_=*α*_1_=*α*).

The true VE against SI is:

$${{\begin{aligned} {VET}_{SI} = 1 - {RRT}_{SI} \quad \text{where} \quad {RRT}_{SI} = \frac{P(Y=2|V=1)}{P(Y=2|V=0)}. \end{aligned}}} $$

The true VE against MAI is:

$$\begin{aligned} {VET}_{MAI} &= 1 - {RRT}_{MAI} \quad \text{where} \quad \\ {RRT}_{MAI} &= \frac{P(Y=2,M=1|V=1)}{P(Y=2,M=1|V=0)}. \end{aligned} $$

Using the parameters defined above, *V* *E* *T*_*SI*_ and *V* *E* *T*_*MAI*_ can be written as:

$$\begin{array}{*{20}l} &{} {VET}_{SI} = 1 - {RRT}_{SI} = 1 - {\frac{\gamma_{10}(1-\pi)+\gamma_{11}\pi}{{\gamma_{00}}(1-\pi)+\gamma_{01}\pi}}\\ &{} {VET}_{MAI} = 1 - {RRT}_{MAI} = 1 - {\frac{\Psi_{F}\left[\gamma_{10}(1-\pi)+\lambda\gamma_{11}\pi\right]}{\gamma_{00}(1-\pi)+\lambda\gamma_{01}\pi}}. \end{array} $$

The proofs of these results can be found in Appendix 1.

### Estimates of VE in our model

In both the TN and TCC study designs, VE is estimated as one minus the odds ratio (OR) in the *C*×*V* table cross-classifying the individuals included in the study, where *C* is a binary indicator of case/control status with *C*=1 for a case. For convenience, the TN and TCC studies will be represented by the letters A and B, respectively. In a TN study, the case/control variable is denoted *C*_*A*_, where (*C*_*A*_=1)=(*M*=1,*T*=1) and (*C*_*A*_=0)=(*M*=1,*T*=0). Then the estimate of VE is: *V* *E*_*A*_=1−*O* *R*_*A*_, where

$${{\begin{aligned} {OR}_{A} = {\frac{P(C_{A}=1,V=1|M=1)P(C_{A}=0,V=0|M=1)}{P(C_{A}=1,V=0|M=1)P(C_{A}=0,V=1|M=1)}}. \end{aligned}}} $$

Note that all the probabilities condition on *M*=1 as only individuals who seek medical care for ARI are included in the TN study.

In a TCC study, the case/control variable is denoted *C*_*B*_. Cases are defined in the same way as in the TN study, i.e., (*C*_*B*_=1)=(*M*=1,*T*=1)=(*C*_*A*_=1). Controls are individuals included in a random sample drawn from all the asymptomatic individuals in the study population. In other words, (*C*_*B*_=0) is a random subset of (*Y*=0). In addition, we define a binary variable *B* indicating whether or not a person is included in the TCC study, i.e., (*B*=1)=(*C*_*B*_=1or*C*_*B*_=0). The VE estimate is based on the OR in the *C*_*B*_×*V* table when all the probabilities condition on *B*=1: *V* *E*_*B*_=1−*O* *R*_*B*_, where

$${} {OR}_{B} = {\frac{P(C_{B}=1,V=1|B=1)P(C_{B}=0,V=0|B=1)}{P(C_{B}=1,V=0|B=1)P(C_{B}=0,V=1|B=1)}}. $$

Note that in a real-life study, the odds ratios are estimated from the relative frequencies of the corresponding events, rather than from their (unknown) probabilities. Therefore, the model-based estimates of VE defined above are actually the expected values of the observed estimates. For convenience we will continue to refer to them as “the VE estimates”.

Using the parameters defined above, *V* *E*_*A*_ and *V* *E*_*B*_ can be written as follows:

$${VE}_{A} = 1 - {\frac{\Psi_{F}\left[\gamma_{10}\alpha_{0}(1-\pi) + \lambda\gamma_{11}\alpha_{1}\pi\right]\left[\beta_{00}(1-\alpha_{0})(1-\pi) + \lambda\beta_{01}(1-\alpha_{1})\pi\right]}{\left[\gamma_{00}(1-\alpha_{0})(1-\pi) + \lambda\gamma_{01}(1-\alpha_{1})\pi\right]\left[\beta_{10}\alpha_{0}(1-\pi) + \lambda\beta_{11}\alpha_{1}\pi\right]}}, $$

$${VE}_{B} = 1 - {\frac{\Psi_{F}\left[\gamma_{10}\alpha_{0}(1-\pi) + \lambda\gamma_{11}\alpha_{1}\pi\right]\left[(1-\gamma_{00}-\beta_{00})(1-\alpha_{0})(1-\pi)+(1-\gamma_{01}-\beta_{01})(1-\alpha_{1})\pi\right]}{\left[\gamma_{00}(1-\alpha_{0})(1-\pi) + \lambda\gamma_{01}(1-\alpha_{1})\pi\right]\left[(1-\gamma_{10}-\beta_{10})\alpha_{0}(1-\pi)+(1-\gamma_{11}-\beta_{11})\alpha_{1}\pi\right]}}. $$

The proofs can be found in Appendix 2.

### Bias and standard errors of estimates

The bias of an estimate of VE is the difference between the expected value of the estimate and the true VE. As the true VE depends on the outcome of interest (SI or MAI), the bias of each estimated VE will be evaluated separately for each of the two outcomes.

In Appendix 3 we use approximations based on the “Delta method” for the standard errors (SEs) of odds ratios [13] to derive expressions for the SEs of both VE estimates in terms of the parameters and the corresponding sample size(s). For evaluating the SEs we consider the observed odds ratios, where the probabilities are replaced by the corresponding observed relative frequencies.

The values of bias reported in the text and tables represent absolute numbers. For example, if the true VE is 60% (i.e., 0.6) and the range of bias (-0.40, -0.20). This means that the estimated VE varies from 0.20 (underestimating the true VE = 0.6 by 0.40) to 0.80 (overestimating the true VE by 0.20).

### Probability ratios

Next, we define a few probability ratios comparing vaccinees and non-vaccinees or healthy and frail individuals. These ratios will be helpful in the presentation of the results (see Table 1 for a full list of the notations used in this paper).

- $\rho _{\beta } = {\frac {\beta _{1x}}{\beta _{0x}}}$, the ratio of the probabilities of NFARI comparing a vaccinated and an unvaccinated person of the same health status.
- $\eta _{\beta } = {\frac {\beta _{v1}}{\beta _{v0}}}$, the ratio of the probabilities of NFARI comparing a healthy and a frail person of the same vaccination status.
- $\rho _{\gamma } = {\frac {\gamma _{1x}}{\gamma _{0x}}}$, the ratio of the probabilities of FARI comparing a vaccinated and an unvaccinated person of the same health status.
- $\eta _{\gamma } = {\frac {\gamma _{v1}}{\gamma _{v0}}}$, the ratio of the probabilities of FARI comparing a healthy and a frail person of the same vaccination status.

The parameters *λ* and *Ψ*_*F*_ defined earlier are also probability ratios:

- $\lambda = {\frac {\delta _{yv1}}{\delta _{yv0}}}$ The ratio of the probabilities of seeking medical care comparing a healthy and a frail person of the same vaccination status. We assume that this ratio is the same for FARI and NFARI patients.
- $\Psi _{F} = {\frac {\delta _{21x}}{\delta _{20x}}}$ The ratio of the probabilities of seeking medical care comparing a vaccinated and an unvaccinated FARI patient of the same health status.

Table 2 presents the main sources of bias that can be identified from our model. The absence of bias A is essential for the validity of the TN design, since the VE estimate from this design is based on comparing the odds of being vaccinated in FARI patients (cases) and NFARI patients (controls). This bias may be a result of virus interference [6] (if vaccinees are more likely than non-vaccinees to contract NFARI, then the estimated VE will be falsely high). Biases B1 and B2 represent the effects of health status on the probabilities of NFARI and FARI, respectively. These effects, which are sometimes called the ‘*healthy vaccinee effect*’, represent the confounding resulting from association of health status with the probability of exposure (vaccination) and the outcome. Bias BS is a special case of *B*1∩*B*2. It results when health status affects both the probabilities of FARI and NFARI but the risk ratios comparing a healthy and a frail person are the same for the both types of ARIs. Bias C represents the effect of vaccination status on the probability of seeking care in patients with SI. This effect may be due to less severe symptoms in vaccinated persons compared to unvaccinated ones. As stated earlier, we assume perfect sensitivity and specificity of the influenza test (*τ*_1_=0, *τ*_2_=1), as it is well-known that misclassifications result in negatively-biased estimates of effectiveness.

**Table 2:** Sources of bias

Source of bias	Description
A	Vaccination affects the probability of NFARI, i.e. *ρ*_*β*_≠1. This may result from virus interference [6].
B1	Health status affects the probability of NFARI, i.e. *η*_*β*_≠1.
B2	Health status affects the probability of FARI, i.e. *η*_*γ*_≠1.
BS	Health status affects the probability of FARI and NFARI, and the risk ratios comparing a healthy and a frail person are the same for both types of ARI, i.e. *η*_*β*_=*η*_*γ*_≠1. This is a special case of $B1 \bigcap B2$.
C	Vaccination affects the probability of seeking medical care for FARI, while it does not affect the probability of seeking medical care for NFARI, i.e. *Ψ*_*F*_≠1. This may result from reduced severity of symptoms in vaccinated influenza patients.
D	Health status affects the probabilities of seeking medical care against FARI and NFARI, i.e. *λ*≠1.

## Results

### Sources of bias

We first state conditions for the unbiasedness of the VE estimate based on the TN design. The proofs of these results can be found in Appendix 4.

- Under random vaccination (*α*_0_=*α*_1_), the estimate of VE when the outcome of interest is SI is unbiased if biases A and C are absent. When the outcome of interest is MAI, the estimate of VE is unbiased if bias A is absent.
- Under non-random vaccination (*α*_0_≠*α*_1_), the estimate of VE when the outcome of interest is SI is unbiased if biases A, B1, B2, and C are absent. When the outcome of interest is MAI, the estimate of VE is unbiased if biases A, B1, and B2 are absent.

It is interesting to note that the absence of any source of bias, the OR-based VE estimate from a TN study is unbiased even if the ‘rare disease’ assumption is not satisfied, while the OR-based estimate from a TCC study is biased. To show this, let’s use the following simplified notation: *α* = probability of being vaccinated, *β* = probability of NFARI, *γ*_0_ and *γ*_1_ = probabilities of FARI in unvaccinated and vaccinated, respectively, and *δ* = probability of seeking care. Then the true VE is 1−*ρ*, where *ρ* = *γ*_1_/ *γ*_0_ is the risk ratio. In a TN study, the probabilities of vaccinated and unvaccinated cases are *α*∗*δ*∗*γ*_1_, and (1−*α*)∗*δ*∗*γ*_0_, respectively. The corresponding probabilities of controls are *α*∗*δ*∗*β*, and (1−*α*)∗*δ*∗*β*, respectively. Then the OR in the table of case-control status by vaccination status equals to *ρ*, i.e. the true risk ratio, implying that the estimated VE is unbiased. In a TCC studies, the probabilities of cases are the same as in the TN study, while the probabilities of vaccinated and unvaccinated controls (individuals without ARI) are *ϕ*∗*α*∗(1−*γ*_1_−*β*) and *ϕ*∗(1−*α*)∗(1−*γ*_0_−*β*), respectively, where *ϕ* is the sampling fraction of controls. Hence, the OR in the TCC study is [*ρ*∗(1−*γ*_0_−*β*)]/(1−*ρ*∗*γ*_0_−*β*). This OR is less than *ρ* (the true RR) if *ρ*> 0, hence the estimated VE exceeds the true VE in a TCC study as long as the true VE is positive.

Next we explore the magnitude of the effects of various sources of bias and their combinations. We consider three scenarios for vaccination probabilities (see Table 3). In Table 4 we present the range and the maximum absolute value of the bias of VE estimates resulting from TN and TCC studies under the three vaccination scenarios and various combinations of sources of bias. For these results we used the following baseline values of some of the parameters: *π*=0.7, *β*_00_=0.2, *γ*_00_=0.1, *δ*_*SN*_=0.2, *δ*_*SF*_=0.3, *ρ*_*γ*_=0.4. *π* is the probability of being ‘healthy’; *β*_00_ and *γ*_00_ are the probabilities of NFARI and FARI, respectively, for an unvaccinated ‘frail’ person; *δ*_*SN*_ and *δ*_*FN*_ are the probabilities of seeking medical care for NFARI and FARI, respectively, for an unvaccinated ‘frail’ person; *ρ*_*γ*_ is the risk ratio comparing the probability of FARI for a vaccinated and an unvaccinated person - thus, the true VE against SI is 1 - 0.4 = 0.6 (60%). The values of *β*’s, *γ*’s are based on data from various randomized placobo-controlled trials (see [14], Table A1), and the values of *δ* are based on data from several observational studies. In all the tables, figures and examples, values of VE are presented as fractions, rather than percentages.

**Table 3:** Three scenarios for vaccination probabilities

Vaccination scenario	Definition
1	Random vaccination, $\alpha _{0}^{\ \mathrm {a}} = \alpha _{1}^{\ \mathrm {b}} = 0.6$
2	Healthy individuals are more likely to be vaccinated than frail individuals: *α*_0_=0.4, *α*_1_=0.8.
3	Healthy individuals are less likely to be vaccinated than frail individuals: *α*_0_=0.8, *α*_1_=0.4.

^a^ *α*_0_ is the probability of vaccination for frail persons

^b^ *α*_1_ is the probability of vaccination for health persons

**Table 4:** Estimate of VE against symptomatic influenza and medically-attended influenza: range of bias and maximum absolute value of bias under various combinations of sources of bias

^a^Sources of bias: A - vaccination affects the probability of non-influenza ARI (NFARI), B1 - health status affects the probability of NFARI, B2 - health status affects the probability of influenza ARI (FARI), BS is a special case of *B*1∩*B*2 where the probabilities of FARI and NFARI depend on health status but the effect of health status on these probabilities is the same for both types of ARI, C - vaccination affects the probability of seeking medical care for FARI while it does not affect the probabilities of seeking care for NFARI, D - health status affects the probabilities of seeking medical care against FARI and NFARI

^b^Scenario: 1 - random vaccination, 2 - healthy person more likely than frail persons to be vaccinated, 3 - healthy person less likely than frail persons to be vaccinated

^c^Bias = estimated VE - true VE. The range of the bias is the interval between the smallest and the largest value of the bias (accounting for the sign) using different combinations of the model parameters. The sign of bias indicates the direction of the difference between the estimated and the true VE. A negative sign corresponds to underestimation while a positive bias indicates overestimation

^d^Maximum absolute value of bias is largest difference between the estimated and the true VE when the sign of the difference in ignored: …Little or no bias (absolute bias less than 0.05),  Moderate bias (absolute bias greater than or equal to 0.05 and less than 0.10),  Substantial bias (absolute bias greater than or equal to 0.10 and less than 0.20),  Severe bias (absolute bias 0.20 or more)

**Example:** Under source of bias A, when the outcome of interest is SI, the TN study (under all vaccination scenarios) has a range of bias of (-0.40, 0.20). This means that the estimated VE varies from 0.20 (underestimating the true VE = 0.6 by 0.40) to 0.80 (overestimating the true VE by 0.20). When the sign of bias is ignored then the greatest difference between the estimated and the true VE is 0.40, hence the maximum absolute value of the bias is 0.40

In the calculations for Tables 4 and 5, when a source of bias was present we used a reasonable range for the corresponding probability ratio. When bias A was present, *ρ*_*β*_ was allowed to vary from 0.5 to 2.0. For biases B1, B2, and BS, we allowed *η*_*β*_ and *η*_*γ*_ to vary between 0.5 and 1.0, since one would not expect frail persons to have lower probabilities of ARI, compared to healthy persons. For bias C, the ratio *Ψ*_*F*_ could vary between 0.5 to 1.0, since one would expect vaccination to reduce the probability that a person with SI will seek medical care compared to a person with ARI resulting from a different pathogen. For bias D, we let *λ* vary between 0.5 to 2.0.

**Table 5:** Minimum, mean and maximum standard errors of VE estimates under various combinations of source of bias

Design	Scenario^b^	Test-negative			Tranditional case-control		
		Min	Mean	Max	Min	Mean	Max
Source of bias^a^							
None	1	0.06	0.06	0.06	0.05	0.05	0.05
	2	0.06	0.06	0.06	0.05	0.05	0.05
	3	0.06	0.06	0.06	0.05	0.05	0.05
A	1	0.03	0.05	0.11	0.04	0.05	0.06
	2	0.03	0.05	0.10	0.04	0.05	0.06
	3	0.03	0.05	0.11	0.04	0.05	0.07
B1	1	0.05	0.05	0.06	0.05	0.05	0.05
	2	0.06	0.06	0.07	0.05	0.05	0.05
	3	0.04	0.05	0.06	0.05	0.05	0.05
B2	1	0.06	0.06	0.06	0.05	0.05	0.05
	2	0.05	0.05	0.06	0.04	0.04	0.05
	3	0.06	0.07	0.08	0.05	0.06	0.06
B1,B2	1	0.05	0.06	0.06	0.05	0.05	0.05
	2	0.05	0.06	0.07	0.04	0.04	0.05
	3	0.04	0.06	0.08	0.05	0.06	0.07
BS	1	0.06	0.06	0.06	0.05	0.05	0.05
	2	0.06	0.06	0.06	0.04	0.05	0.04
	3	0.06	0.06	0.06	0.05	0.07	0.06
C	1	0.03	0.04	0.06	0.03	0.04	0.05
	2	0.03	0.04	0.06	0.03	0.04	0.05
	3	0.03	0.05	0.06	0.03	0.04	0.05
D	1	0.06	0.06	0.06	0.05	0.05	0.05
	2	0.06	0.06	0.06	0.04	0.05	0.06
	3	0.06	0.06	0.06	0.04	0.05	0.06
C,D	1	0.03	0.04	0.06	0.03	0.04	0.05
	2	0.03	0.04	0.06	0.02	0.04	0.06
	3	0.03	0.05	0.06	0.02	0.04	0.06
B1,B2,C,D	1	0.03	0.04	0.06	0.03	0.04	0.05
	2	0.03	0.04	0.07	0.02	0.03	0.06
	3	0.02	0.05	0.08	0.02	0.04	0.09
BS,C,D	1	0.03	0.06	0.04	0.03	0.05	0.04
	2	0.03	0.06	0.04	0.02	0.06	0.03
	3	0.03	0.06	0.04	0.02	0.09	0.04
A,B1,B2,C,D	1	0.02	0.04	0.11	0.02	0.04	0.07
	2	0.02	0.04	0.14	0.01	0.04	0.08
	3	0.01	0.04	0.15	0.02	0.05	0.12

^a^Source of bias: A: Vaccination affects the probability of NFARI

B1: Health status affects the probability of NFARI

B2: Health status affects the probability of FARI

BS: Health status affects the probability of FARI and NFARI, and the risk ratios comparing a healthy and a frail person are the same for both types of ARI

C: Vaccination affects the probability of seeking medical care for FARI, while it does not affect the probability of seeking medical care for NFARI

D: Health status affects the probabilities of seeking medical care against FARI and NFARI

^b^Vaccination Scenarios

1: Random vaccination

2: Healthy person more likely than frail persons to be vaccinated

3: Healthy person less likely than frail persons to be vaccinated

For each combination of two or more sources of bias, we calculated the minimum, mean, and maximum of the bias and the absolute values of the bias by allowing the probability ratios that are not fixed to vary independently in the ranges specified above. For example, when biases A, B1, and B2 are absent, we used *ρ*_*β*_=*η*_*β*_=*η*_*γ*_=1,0.5≤*Ψ*_*F*_≤1,0.5≤*λ*≤2.

## Summary of results

### The impact of sources of bias

Our model allows us to evaluate the impact of the sources of bias listed in Table 2. Each source of bias is a result of a possible effect of vaccination or health status on the probability of FARI or NFARI or seeking care. Below we summarize our results for each of the sources of bias. We also use numerical examples to illustrate the magnitude and direction associated with each source of bias. *Unless otherwise specified, the true VEs against SI and MAI are 0.6 (60%).* In each of these examples we assume that only one source of bias is present.

**Fig. 2:**
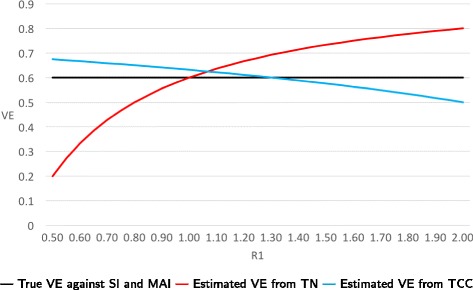
True and estimated VEs as a function of R1 = P(NFARI if vaccinated)/P(NFARI if unvaccinated) when only bias A is present

**Fig. 3:**
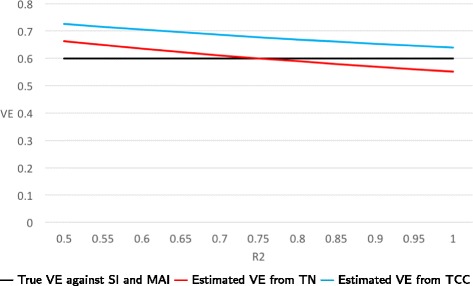
True and estimated VE’s when only biases B1 and B2 are present. We set the risk ratio P(NFARI if healthy)/P(NFARI if frail) =0.75 and let the risk ratio R2 = P(FARI if healthy)/P(FARI if frail) vary between 0.5 to 1.0. The probabilities of vaccination are 0.4 and 0.8 for healthy and frail persons, respectively

**Fig. 4:**
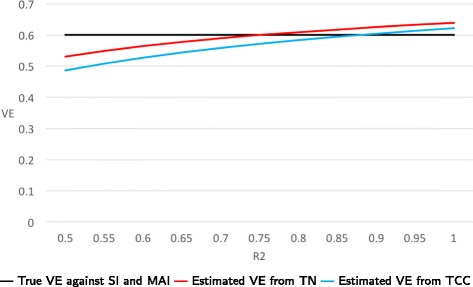
True and estimated VEs when only biases B1 and B2 are present. We set the risk ratio P(NFARI if healthy)/P(NFARI if frail) =0.75 and let the risk ratio R2 = P(FARI if healthy)/P(FARI if frail) vary between 0.5 to 1.0. The probabilities of vaccination are 0.8 and 0.4 for healthy and frail persons, respectively

**Fig. 5:**
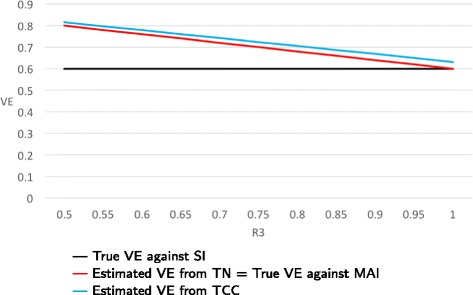
True and estimated VEs when only bias C is present as function of R3 = P(seeking medical care against FARI if vaccinated)/P(seeking medical care against FARI if unvaccinated)

**Fig. 6:**
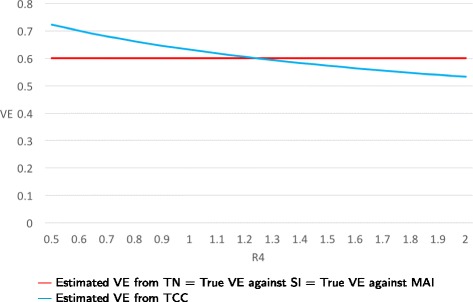
True and estimated VEs when only bias D is present as function of R4 = P(seeking medical care if healthy)/P(seeking medical care if frail). Probabilities of vaccination are 0.8 and 0.4 for healthy and frail persons, respectively

**Fig. 7:**
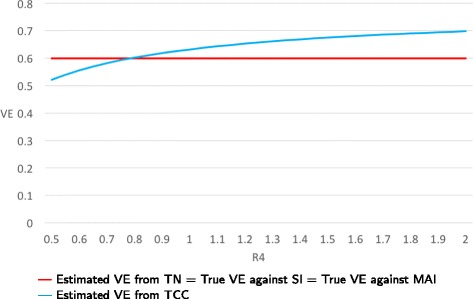
True and estimated VEs when only bias D is present as function of R4 = P(seeking medical care if healthy)/P(seeking medical care if frail). Probabilities of vaccination are 0.4 and 0.8 for healthy and frail persons, respectively

1. **Vaccination affects the probability of NFARI (bias A)**
  This bias does not depend on vaccination scenario nor on the outcome of interest (SI or MAI).
  Estimates of VE from TN studies may suffer from severe bias.
  This effect also affects the bias of VE estimates from TCC studies, though to a lesser extent.
**Example:** As the ratio of the probability of NFARI comparing vaccinated and unvaccinated persons varies from 0.5 to 2.0, VE estimates from TN studies range from 0.2 to 0.8, respectively, while VE estimates from TCC studies range from 0.67 to 0.50, respectively (Fig. 2).

(2) **Health status affects the probabilities of FARI and NFARI (biases B1, B2 - the ‘** ***healthy vaccinee effect*** **’)**
  The bias does not depend on the outcome of interest (SI or MAI).
  Under non-random vaccination, these effects may result in substantial bias of VE estimates from TN or TCC studies. However, this bias is usually less severe compared to the biases resulting from sources A, C and D.
  If the effect of health status on the probability of ARI is the same for FARI and NFARI, i.e., bias BS is present, then the TN-based estimates of VE are unbiased.
**Example:** Suppose that the probabilities of vaccination are 0.8 and 0.4 for healthy and frail persons, respectively. Consider three cases regarding the *risk ratios* P(ARI in a healthy person) / P(ARI in a frail person): (a) When these risk ratios are 0.5 for NFARI and 0.8 for FARI, then the estimated VEs from TN and TCC studies are 0.51 and 0.67, respectively. (b) When the risk ratios are 0.8 for NFARI and 0.5 for FARI, then estimated VEs from TN and TCC studies are 0.67 and 0.72, respectively. (c) When the risk ratios for NFARI and FARI are equal and their common value ranges from 0.5 to 1.0, then estimated VEs from TN studies are always unbiased (i.e., they equal 0.6), while estimates from TCC studies range from 0.63 to 0.73. In Figs. 3 and 4, we set the risk ratio for NFARI to 0.75 and let the risk ratio for FARI vary between 0.5 to 1.0.

(3) **Vaccination affects the probability of seeking medical care for FARI, but it does not affect the probability of seeking care for NFARI (bias C)**
  When this effect is present then the true VEs against SI and MAI may be different as the vaccine directly affects (reduces) the probability of seeking care in influenza cases, but not in controls. Thus the estimates’ bias may depend on the outcome of interest.
  If all other sources of bias are absent, the bias of VE estimates does not depend on the vaccination scenario.
  Estimates of VE from TN or TCC studies may be severely biased when the outcome of interest is SI.
  When the outcome of interest is MAI, estimates of VE from TN studies are unbiased, while the bias of estimates from TCC studies is usually small and is not affected by the magnitude of the effect underlying this source of bias.
**Example:** Let the ratio R = P(seeking medical care against FARI if vaccinated) / P(seeking medical care against FARI if unvaccinated) vary from 0.5 to 1.0. Then the true VE against SI remains fixed at 0.6, while the true VE against MAI varies with R from 0.8 to 0.6. The estimated VEs from TN studies equal the true VE against MAI for all values of R, while the estimated VEs from TCC studies vary from 0.82 to 0.63 (see Fig. 5). For example, when *R*=0.5 then the true VE against MAI is 0.80, and the VE estimates from TN and TCC studies are 0.80 and 0.82, respectively. This translates into severe bias when the outcome of interest is SI but small bias when the outcome of interest is MAI.

(4) **Health status affects the probabilities of seeking care against FARI and NFARI (bias D)**
  The bias of VE estimates does not depend on the outcome of interest (SI or MAI).
  In the absence of other sources of bias, VE estimates from TN studies are unbiased regardless of the vaccination scenario.
  Under non-random vaccination, this effect may result in substantial bias in VE estimates from TCC studies.
**Example:** We assume that the probabilities of seeking care do not depend on vaccination status. As the ratio of the probabilities of seeking care comparing healthy and frail individuals varies from 0.5 to 2.0, VE estimates from TN studies remain fixed at 0.6 (i.e., they are unbiased) under both random and non-random vaccination. When the probabilities of vaccination are 0.8 and 0.4 for healthy and frail persons, respectively, the VE estimates from TCC studies vary from 0.72 to 0.53 (Figs. 6 and 7).

In addition, we found that in some cases the true VEs against SI and MAI are different. Hence, the bias of VE estimates may depend on the outcome against which the vaccine is supposed to protect. For example, if the only sources of bias are BS, C, and D then, the VE estimate from TN studies is unbiased when considering effectiveness against MAI. The same estimate may overestimate the true VE against SI by 0.20 (i.e. 20%).

### Comparison of the bias of VE estimates from TN and TCC studies:

- If one is concerned that vaccination may affect the probability of non-influenza ARI, then one should prefer the TCC study design. However, TCC-based VE estimates may still be biased in this case. For example, when the ratio of the probability of NFARI comparing a vaccinated and an unvaccinated person is 0.5, then the bias of VE estimate from TN study is -0.4 while the bias of VE estimate from TCC study is 0.07.
- Under non-random vaccination, effects of health status on probabilities of influenza and non-influenza ARI (the ‘*healthy vaccinee effect*’) may bias VE estimates from both study designs. In general, TN-based estimates perform slightly better than TCC-based estimates when this effect is believed to be the main source of bias. If the effect of health status is similar for FARI and NFARI, then the TN design produces less biased estimates compared to the TCC design. For example, suppose the probabilities of vaccination are 0.4 and 0.8 for healthy and frail persons, respectively. When the risk ratios for NFARI and FARI are both 0.75, then the VE estimate from TN study is unbiased, while the bias of VE estimate from TCC study is 0.07.
- If one assumes that vaccination does not affect the probability of non-influenza ARI but one is concerned that vaccinated influenza patients are less likely to seek care than unvaccinated patients (because of reduced symptoms severity), then VE estimates may suffer from severe bias in both study designs when the outcome of interest is SI. In this case, the bias of TN-based estimates may be somewhat smaller than that of TCC-based estimates. This source of bias does not affect VE estimates when the outcome of interest is MAI! For example, suppose that the ratio comparing vaccinated and unvaccinated FARI cases w.r.t. the probability of seeking medical care is 0.5. When the outcome of interest is SI, then the bias of a VE estimate from TN study is 0.2 and the bias of a VE estimate from TCC study is 0.22. When the outcome of interest is MAI, then the VE estimate from a TN study is unbiased, while the bias of a VE estimate from a TCC study is 0.02.
- Under non-random vaccination, the TN study design is preferable to the TCC design if one is concerned about bias resulting from possible effect of a person’s health status on her/his probability of seeking care against ARI. For example, suppose that the probabilities of vaccination are 0.8 and 0.4 for healthy and frail persons, respectively. When the ratio of the probabilities of seeking medical care comparing healthy and frail persons is 0.5, then the VE estimate from TN study is unbiased while the bias of VE estimate is 0.12.

### Precision of VE estimates

Table 5 presents the standard errors of VE estimates from TN and TCC studies. From this table we conclude that:

- Non-random vaccination may reduce precision of VE estimates.
- If the probability of NFARI is associated with vaccination status, then VE estimates from TN studies are somewhat less precise compared to VE estimates from TCC studies, although the differences in precision were small.
- If the probability of NFARI is not associated with vaccination status, then the precision of VE estimates from TN and TCC studies are similar.

## Discussion and conclusions

We developed a new model for the evaluation of the bias and precision of influenza estimates from case-control studies. The new model is more comprehensive than previously suggested models [5, 14–18] for the following reasons:

- It allows assessment of the impact of non-random vaccination.
- It incorporates a confounder (health status) which links vaccination status with the probabilities of ARI and of seeking medical care for these ARIs.
- By including parameters corresponding to the probabilities of seeking medical care, the model allows us to examine the effect of association of these probabilities with vaccination and health status on the bias of VE estimates.
- The model allows evaluating and comparing the precision of VE estimates.

Some of the sources of bias discussed here have been identified and addressed in earlier publications, but, to our best knowledge, none of the previous papers present a comprehensive discussion of all the possible sources of bias that may arise under a given model. In addition, the current model attributes the associations between the various factors involved in estimation of VE (vaccination, contracting influenza and non-influenza ARIs and seeking medical care) to an underlying covariate. Previously published models, including an earlier version of our model [14], included parameters representing these associations but these associations were not based on a common underlying factor. Therefore, we believe that the current results and conclusions may differ from those derived from less structured models.

Our calculations confirm earlier findings [15] that when the probability of non-inluenza ARI depends on vaccination status, VE estimates from test-negative studies may be severely biased. However, even when this probability is not affected by vaccination, VE estimates from the two types of case-control studies considered in this work may suffer from substantial bias. In addition to the well-known ‘*healthy vaccinee effect*’ (probabilities of vaccination and of ARI depend on health status), bias of VE estimates may result from heterogeneities in health-care-seeking behaviors. Specifically, if vaccination reduces the probability that an influenza patient seeks medical care (because her/his symptoms are less severe than those of an unvaccinated influenza patient) then VE estimates from TN or TCC studies may grossly overestimate the true VE against SI. On the other hand, when the outcome of interest is MAI then the biases resulting from vaccine-related reduction in symptoms’ severity are very small. Recent papers [7–9] found evidence of vaccine-associated reduction in influenza patient’s symptoms severity. The effects of health-care-seeking behaviors on VE estimates from studies in which only ARI patients who seek medical care may become cases need to be further investigated.

The results of this work lead to the following conclusions:

- In general, estimates of influenza VE from case-control studies where only ARI patients seeking medical care are tested for influenza infection may suffer from severe bias, i.e. an absolute bias of 20% or more, especially when the outcome of interest is SI.
- The bias of VE estimates may depend on the outcome against which the vaccine is supposed to protect. When the outcome of interest is MAI, seeking medical care is a component of the outcome. In other words, the true VE against MAI reflects the vaccine effect on seeking medical care and on contracting influenza. This explains why true VE against MAI may differ from true VE against SI. When bias C is present, the vaccine directly affects (reduces) the probability of seeking care in influenza cases, but not in controls. As a result, VE against MAI is lower than VE against SI.
- Influenza VE estimates from TN studies are usually presented as ‘VE against medically-attended influenza’. However, the media and lay persons may interpret these VE estimates simply as the protective effectiveness of vaccination against contracting influenza illness, i.e. VE against SI. Health authorities and the public should be made aware of this distinction.
- When the outcome of interest is SI, the TN design provides valid estimates (i.e., no or small bias) if the following assumptions are satisfied: (a) vaccination does not affect the probability of non-influenza ARI, (b) effects of confounding variables on the probabilities of influenza and non-influenza ARI are similar, and (c) vaccination does not affect the probabilities of seeking medical care for influenza ARI due to reduced severity of symptoms. When the outcome of interest is MAI, then only assumptions (a) and (b) are necessary for obtaining a valid VE estimate from a TN study.
- Estimates of VE from TCC studies have small bias when the outcome of interest is SI if assumptions (a) and (c) are satisfied, assumption (b) is replaced by the stronger assumption (b*) of no presence of confounding, and the additional assumption (d) that the probabilities of seeking medical care for ARI are not affected by potential confounders is satisfied. When the outcome of interest is MAI, then TCC-based estimates of VE have small bias under assumptions (a), (b*), and (d).
- It is important to collect more data on health-care-seeking behaviors of ARI patients and to study the effects of vaccination and potential confounders on these behaviors.

In summary, the test negative design produces less biased VE estimates, compared to the traditional case-control design provided that vaccination does not modify the probability of non-influenza ARI. However, this very popular study design may still produce biased estimates of influenza VE, especially when the outcome of interest is symptomatic influenza. One can expect *monitored cohort studies*, where every study participant reporting an ARI is tested for influenza infection, to provide less biased estimates of VE against SI. In a future publication we plan to compare the bias of these cohort studies, which are much more expensive, with that of TN studies.

Our study has a few limitations:

- In order to focus on bias associated with the study designs, we ignored bias resulting from misclassification of infection and vaccination status.
- Our model does not account for the dynamics of outbreaks of influenza and other ARI-causing infections.
- We only consider unadjusted VE estimates as we tried to focus on sources of bias rather than on how one can reduce bias using standard or novel statistical techniques [19].

In the future we plan to improve the model by incorporating dynamics of the related processes. We also plan to use stochastic simulations to assess bias and precision of influenza VE estimates for other study designs (e.g. cohort studies) and to propose new study designs resulting in less biased VE estimates.

## Appendix 1

### True VE‘s in our model

The true VE against SI is:

$${} \begin{aligned} {VET}_{SI} = 1 - {RRT}_{SI} \quad \text{where} \quad {RRT}_{SI} = {{P(Y=2|V=1)}\over {P(Y=2|V=0)}}. \end{aligned} $$

Since

$${{\begin{aligned} & P(Y=y|V=v) = \sum_{x} P(Y=y|V=v,X=x)P(X=x|V=v)\\ & P(V=v) = \sum_{x} P(V=v|X=x)P(X=x)\\ & P(X=x|V=v) = {P(V=v|X=x)P(X=x)\over P(V=v)}\\ & \quad\;\; = {P(V=v|X=x)P(X=x)\over {\sum_{x} P(V=v|X=x)P(X=x)}}\\ \end{aligned}}} $$

we have

$${{\begin{aligned} P(X=0|V=0) &={P(V=0|X=0)P(X=0)\over {\sum_{x} P(V=0|X=x)P(X=x)}}\\ &= {{(1-\alpha_{0})(1-\pi)}\over{(1-\alpha_{0})(1-\pi)+(1-\alpha_{1})\pi}} = 1-\pi,\\ P(X=0|V=1) &={P(V=1|X=0)P(X=0)\over {\sum_{x} P(V=1|X=x)P(X=x)}} \\ &= {{\alpha_{0}(1-\pi)}\over{\alpha_{0}(1-\pi)+\alpha_{1}\pi}}=1-\pi,\\ P(X=1|V=0) &={P(V=0|X=1)P(X=1)\over {\sum_{x} P(V=0|X=x)P(X=x)}} \\&= {{(1-\alpha_{1})\pi}\over{(1-\alpha_{0})(1-\pi)+(1-\alpha_{1})\pi}}=\pi,\\ P(X=1|V=1) &={P(V=1|X=1)P(X=1)\over {\sum_{x} P(V=1|X=x)P(X=x)}} \\&= {{\alpha_{1}\pi}\over{\alpha_{0}(1-\pi)+\alpha_{1}\pi}}=\pi.\\ \end{aligned}}} $$

Since, for true VE, we have: *α*_0_=*α*_1_. We can get,

$${{\begin{aligned} P(Y&=2|V=1) = \sum_{x} P(Y=2|V=1,X=x)P(X=x|V=1)\\ &= P(Y=2|V=1,X=0)P(X=0|V=1)\\&\quad+P(Y=2|V=1,X=1)P(X=1|V=1)\\ &= \gamma_{10}(1-\pi)+ \gamma_{11}\pi \end{aligned}}} $$

$${{\begin{aligned} P(Y&=2|V=0) = \sum_{x} P(Y=2|V=0,X=x)P(X=x|V=0)\\ &= P(Y=2|V=0,X=0)P(X=0|V=0)\\&\quad+P(Y=2|V=0,X=1)P(X=1|V=0)\\ &= \gamma_{00}(1-\pi)+\gamma_{01}\pi \end{aligned}}} $$

So that,

$$\begin{array}{*{20}l} {RRT}_{SI} &= {{P(Y=2|V=1)}\over {P(Y=2|V=0)}} = {{\gamma_{10}(1-\pi)+\gamma_{11}\pi}\over{\gamma_{00}}(1-\pi)+\gamma_{01}\pi} \end{array} $$

Therefore,

$$\begin{array}{*{20}l}{} {VET}_{SI} = 1 - {RRT}_{SI} &= 1 - {{\gamma_{10}(1-\pi)+\gamma_{11}\pi}\over{\gamma_{00}}(1-\pi)+\gamma_{01}\pi} \quad \quad \text{Q.E.D.} \end{array} $$

The true VE against MAI is:

$$\begin{aligned} &{VET}_{MAI} = 1 - {RRT}_{MAI} \quad \text{where} \quad\\ &{RRT}_{MAI} = {{P(Y=2,M=1|V=1)}\over {P(Y=2,M=1|V=0)}}. \end{aligned} $$

Since

$${{\begin{aligned} &P(Y=2,M=1|V=v,X=x) = P(M=1|Y=2,\\& \quad\! V=v,X=x)*P(Y=2|V=v,X=x)\\ &P(Y=2,M=1|V=v) \\& \qquad\!\! = \sum_{x} P(Y=2,M=1|V=v,X=x)P(X=x|V=v) \end{aligned}}} $$

we can get,

$${{\begin{aligned} P(Y&=2,M=1|V=1) \\&= \sum_{x} P(Y=2,M=1|V=1,X=x)P(X=x|V=1)\\ &=\delta_{210}\gamma_{10}(1-\pi)+\delta_{211}\gamma_{11}\pi\\ P(Y&=2,M=1|V=0) \\&= \sum_{x} P(Y=2,M=1|V=0,X=x)P(X=x|V=0)\\ &=\delta_{200}\gamma_{00}(1-\pi)+\delta_{201}\gamma_{01}\pi\\ \end{aligned}}} $$

Therefore,

$$\begin{array}{*{20}l} {RRT}_{MAI}&= {{P(Y=2,M=1|V=1)}\over {P(Y=2,M=1|V=0)}}\\ &={{\delta_{210}\gamma_{10}(1-\pi)+\delta_{211}\gamma_{11}\pi}\over{\delta_{200}\gamma_{00}(1-\pi)+\delta_{201}\gamma_{01}\pi}}\\ &={{\delta_{SF}\Psi_{F}\gamma_{10}(1-\pi)+\delta_{SF}\Psi_{F}\lambda\gamma_{11}\pi}\over{\delta_{SF}\gamma_{00}(1-\pi)+\delta_{SF}\lambda\gamma_{01}\pi}}\\ &= {{\Psi_{F}[\gamma_{10}(1-\pi)+\lambda\gamma_{11}\pi]}\over{\gamma_{00}(1-\pi)+\lambda\gamma_{01}\pi}}\\ \end{array} $$

Hence,

$$\begin{array}{*{20}l} {VET}_{MAI} &= 1 - {RRT}_{MAI} \\ &= 1 - {{\Psi_{F}\left[\gamma_{10}(1-\pi)+\lambda\gamma_{11}\pi\right]}\over{\gamma_{00}(1-\pi)+\lambda\gamma_{01}\pi}}. \quad \quad \text{Q.E.D.} \end{array} $$

## Appendix 2

### Model-based estimates of VE

The model-based estimate from TN study is:

$${{\begin{aligned} {VE}_{A} &= 1 - {OR}_{A},\quad \text{where}\quad\\ {OR}_{A} &= {{P(C_{A}=1,V=1|M=1)P(C_{A}=0,V=0|M=1)}\over{P(C_{A}=1,V=0|M=1)P(C_{A}=0,V=1|M=1)}}. \end{aligned}}} $$

*O* *R*_*A*_ can be written as:

$${} {OR}_{A} = {{P(M=1,T=1,V=1)P(M=1,T=0,V=0)}\over{P(M=1,T=1,V=0)P(M=1,T=0,V=1)}} $$

$${{\begin{aligned} P(M&=1,T=1,V=v) = \sum_{x} P(M=1,T=1,\\&\quad V=v|X=x)P(X=x)\\ &=\sum_{x} P(T=1|M=1,V=v,X=x)\\&\quad P(M=1,V=v|X=x)P(X=x)\\ &=\sum_{x} P(Y=2|M=1,V=v,X=x)\\&\quad P(M=1|V=v,X=x)P(V=v|X=x)P(X=x) \\ &= \sum_{x} P(M=1|Y=2,V=v,X=x)\\&\quad P(Y=2|V=v,X=x)P(V=v|X=x)P(X=x) \end{aligned}}} $$

$${{\begin{aligned} P(M&=1,T=0,V=v) = \sum_{x} P(M=1,T=0,\\&\quad V=v|X=x)P(X=x)\\ &=\sum_{x} P(T=0|M=1,V=v,X=x)\\&\quad P(M=1,V=v|X=x)P(X=x)\\ &=\sum_{x} P(Y=1|M=1,V=v,X=x)\\&\quad P(M=1|V=v,X=x)P(V=v|X=x)P(X=x) \\ &= \sum_{x} P(M=1|Y=1,V=v,X=x)\\&\quad P(Y=1|V=v,X=x)P(V=v|X=x)P(X=x) \end{aligned}}} $$

So that,

$$\begin{array}{*{20}l} P(M=1,T=1,V=0) &= \delta_{200}\gamma_{00}(1-\alpha_{0})(1-\pi) + \delta_{201}\gamma_{01}(1-\alpha_{1})\pi\\ &= \delta_{SF}\left[\gamma_{00}(1-\alpha_{0})(1-\pi) + \lambda\gamma_{01}(1-\alpha_{1})\pi\right]\\ P(M=1,T=1,V=1) &= \delta_{210}\gamma_{10}\alpha_{0}(1-\pi) + \delta_{211}\gamma_{11}\alpha_{1}\pi\\ &= \delta_{SF}\Psi_{F}[\gamma_{10}\alpha_{0}(1-\pi) + \lambda\gamma_{11}\alpha_{1}\pi]\\ P(M=1,T=0,V=0) &= \delta_{100}\beta_{00}(1-\alpha_{0})(1-\pi) + \delta_{101}\beta_{01}(1-\alpha_{1})\pi\\ &= \delta_{SN}[\beta_{00}(1-\alpha_{0})(1-\pi) + \lambda\beta_{01}(1-\alpha_{1})\pi]\\ P(M=1,T=0,V=1) &= \delta_{110}\beta_{10}\alpha_{0}(1-\pi) + \delta_{111}\beta_{11}\alpha_{1}\pi\\ &= \delta_{SN}[\beta_{10}\alpha_{0}(1-\pi) + \lambda\beta_{11}\alpha_{1}\pi]\\ \end{array} $$

Therefore,

$${VE}_{A} = 1 - {{\Psi_{F}\left[\gamma_{10}\alpha_{0}(1-\pi) + \lambda\gamma_{11}\alpha_{1}\pi\right]\left[\beta_{00}(1-\alpha_{0})(1-\pi) + \lambda\beta_{01}(1-\alpha_{1})\pi\right]}\over{\left[\gamma_{00}(1-\alpha_{0})(1-\pi) + \lambda\gamma_{01}(1-\alpha_{1})\pi\right]\left[\beta_{10}\alpha_{0}(1-\pi) + \lambda\beta_{11}\alpha_{1}\pi\right]}}. \quad \quad \text{Q.E.D.} $$

The model-based estimates from TCC study is:

$${} {{\begin{aligned} {VE}_{B} &= 1 - {OR}_{B},\quad \text{where}\quad \\ {OR}_{B} &= {{P(C_{B}=1,V=1|B=1)P(C_{B}=0,V=0|B=1)}\over{P(C_{B}=1,V=0|B=1)P(C_{B}=0,V=1|B=1)}}. \end{aligned}}} $$

*O* *R*_*B*_ can be written as:

$${OR}_{B} = {{P(M=1,T=1,V=1)P(Y=0,V=0)}\over{P(M=1,T=1,V=0)P(Y=0,V=1)}}. $$

Since,

$${} {{\begin{aligned} P(Y=0,V=v) &= \sum_{x} P(Y=0,V=v|X=x)P(X=x) \\ &= \sum_{x} P(Y=0|V=v,X=x)\\&\qquad\;\;\; P(V=v|X=x)P(X=x) \end{aligned}}} $$

and *P*(*Y*=0|*V*=*v*,*X*=*x*)=1−*P*(*Y*=1|*V*=*v*,*X*=*x*)−*P*(*Y*=2|*V*=*v*,*X*=*x*)=1−*γ*_*vx*_−*β*_*vx*_, so we have:

$$\begin{array}{*{20}l} P(Y=0,V=0) & = (1-\gamma_{00}-\beta_{00})(1-\alpha_{0})(1-\pi)\\&\quad +(1-\gamma_{01}-\beta_{01})(1-\alpha_{1})\pi\\ P(Y=0,V=1) & = (1-\gamma_{10}-\beta_{10})\alpha_{0}(1-\pi)\\&\quad +(1-\gamma_{11}-\beta_{11})\alpha_{1}\pi \end{array} $$

Therefore,

$${VE}_{B} = 1 - {{\Psi_{F}\left[\gamma_{10}\alpha_{0}(1-\pi) + \lambda\gamma_{11}\alpha_{1}\pi\right]\left[(1-\gamma_{00}-\beta_{00})(1-\alpha_{0})(1-\pi)+(1-\gamma_{01}-\beta_{01})(1-\alpha_{1})\pi\right]}\over{\left[\gamma_{00}(1-\alpha_{0})(1-\pi) + \lambda\gamma_{01}(1-\alpha_{1})\pi\right]\left[(1-\gamma_{10}-\beta_{10})\alpha_{0}(1-\pi)+(1-\gamma_{11}-\beta_{11})\alpha_{1}\pi\right]}}.\quad \quad \text{Q.E.D.} $$

## Appendix 3

### Standard errors of the VE estimates

For TN study, the approximate standard error of *V* *E*_*A*_ is:

$$\begin{array}{*{20}l} SE({VE}_{A}) &= SE({OR}_{A}) \approx {OR}_{A}*SE(log({OR}_{A})) \\ &\approx {{p^{A}_{11}\left(1-p^{A}_{01}\right)}\over{p^{A}_{01}\left(1-p^{A}_{11}\right)}}\sqrt{{1\over{N^{A}}} \left[{1\over{P^{A}_{V1}P^{A}_{11}}}+{1\over{\left(1-P^{A}_{V1}\right)P^{A}_{01}}}+{1\over{P^{A}_{V1}}\left(1-P^{A}_{11}\right)}+{1\over{\left(1-P^{A}_{V1}\right)\left(1-P^{A}_{01}\right)}}\right]} \end{array} $$

where *N*_*A*_ is the number of persons who were tested for influenza (M=1), i.e., the total sample size for the TN study. The probabilities ($p^{A}_{V1}$, $p^{A}_{01}$, $p^{A}_{11}$) can be written in terms of the parameters defined earlier.

Base on what we got earlier, we know

$$\begin{aligned} P(M&=1,V=1) = \sum_{t} P(M=1,V=1,T=t) \\ &= \delta_{110}\beta_{10}\alpha_{0}(1-\pi) + \delta_{111}\beta_{11}\alpha_{1}\pi + \delta_{210}\gamma_{10}\alpha_{0}(1-\pi) + \delta_{211}\gamma_{11}\alpha_{1}\pi\\ &=\alpha_{0}(1-\pi)\left[\delta_{SN}\beta_{10}+\delta_{SF}\Psi_{F}\gamma_{10}\right] + \alpha_{1}\pi\lambda \left[\delta_{SN}\beta_{11}+\delta_{SF}\Psi_{F}\gamma_{11}\right] \end{aligned} $$

$$\begin{aligned} P(M&=1,V=0) = \sum_{t} P(M=1,V=0,T=t) \\ &= \delta_{100}\beta_{00}(1-\alpha_{0})(1-\pi) + \delta_{101}\beta_{01}(1-\alpha_{1})\pi + \delta_{200}\gamma_{00}(1-\alpha_{0})(1-\pi) + \delta_{201}\gamma_{01}(1-\alpha_{1})\pi\\ &= (1-\alpha_{0})(1-\pi)\left[\delta_{SN}\beta_{00}+\delta_{SF}\gamma_{00}\right]+(1-\alpha_{1})\pi\lambda\left[\delta_{SN}\beta_{01}+\delta_{SF}\gamma_{01}\right] \end{aligned} $$

Thus,

$$\begin{array}{*{20}l} P(M&=1) = \sum_{v} P(M=1, V=v)\\ &= (1-\alpha_{0})(1-\pi)\left[\delta_{SN}\beta_{00}+\delta_{SF}\gamma_{00}\right]+(1-\alpha_{1})\pi\lambda \left[\delta_{SN}\beta_{01}+\delta_{SF}\gamma_{01}\right]\\ &\quad + \alpha_{0}(1-\pi) \left[\delta_{SN}\beta_{10}+\delta_{SF}\Psi_{F}\gamma_{10}\right] + \alpha_{1}\pi\lambda \left[\delta_{SN}\beta_{11}+\delta_{SF}\Psi_{F}\gamma_{11}\right] \\ &= (1-\pi) \left[(1-\alpha_{0})(\delta_{SN}\beta_{00}+\delta_{SF}\gamma_{00})+\alpha_{0}(\delta_{SN}\beta_{10}+\delta_{SF}\Psi_{F}\gamma_{10})\right] \\ & \quad + \pi\lambda \left[((1-\alpha_{1}))(\delta_{SN}\beta_{01}+\delta_{SF}\gamma_{01}) + \alpha_{1}(\delta_{SN}\beta_{11}+\delta_{SF}\Psi_{F}\gamma_{11})\right] \end{array} $$

Therefore, we have

$${\begin{aligned} P^{A}_{V1} &= P(V=1|M=1) = {{P(V=1,M=1)}\over{P(M=1)}}\\ &= {{\alpha_{0}(1-\pi)\left[\delta_{SN}\beta_{10}+\delta_{SF}\Psi_{F}\gamma_{10}\right] + \alpha_{1}\pi\lambda\left[\delta_{SN}\beta_{11}+\delta_{SF}\Psi_{F}\gamma_{11}\right]}\over{\tiny{(1-\pi)\left[(1-\alpha_{0})(\delta_{SN}\beta_{00}+\delta_{SF}\gamma_{00})\!+ \alpha_{0}(\delta_{SN}\beta_{10}+\delta_{SF}\Psi_{F}\gamma_{10})\right] + \pi\lambda \left[((1-\alpha_{1}))(\delta_{SN}\beta_{01}+\delta_{SF}\gamma_{01}) + \alpha_{1}(\delta_{SN}\beta_{11}+\delta_{SF}\Psi_{F}\gamma_{11})\right]}}} \end{aligned}} $$

$$\begin{array}{*{20}l} p^{A}_{01} &= {{P(M=1,T=1|V=0)}\over{P(M=1|V=0)}} = {{P(M=1,T=1,V=0)}\over{P(M=1,V=0)}} \\ & = {{\delta_{200}\gamma_{00}(1-\alpha_{0})(1-\pi) + \delta_{201}\gamma_{01}(1-\alpha_{1})\pi}\over{\delta_{100}\beta_{00}(1-\alpha_{0})(1-\pi) + \delta_{101}\beta_{01}(1-\alpha_{1})\pi + \delta_{200}\gamma_{00}(1-\alpha_{0})(1-\pi) + \delta_{201}\gamma_{01}(1-\alpha_{1})\pi}}\\ &={{\delta_{SF} \left[\gamma_{00}(1-\alpha_{0})(1-\pi) + \lambda\gamma_{01}(1-\alpha_{1})\pi\right]}\over{(1-\alpha_{0})(1-\pi) \left[\delta_{SN}\beta_{00}+\delta_{SF}\gamma_{00}\right]+(1-\alpha_{1})\pi\lambda \left[\delta_{SN}\beta_{01}+\delta_{SF}\gamma_{01}\right]}} \end{array} $$

$$\begin{array}{*{20}l} P^{A}_{11} &= {{P(M=1,T=1|V=1)}\over{P(M=1|V=1)}} = {{P(M=1,T=1,V=1)}\over{P(M=1,V=1)}}\\ &= {{\delta_{210}\gamma_{10}\alpha_{0}(1-\pi) + \delta_{211}\gamma_{11}\alpha_{1}\pi}\over{\delta_{110}\beta_{10}\alpha_{0}(1-\pi) + \delta_{111}\beta_{11}\alpha_{1}\pi + \delta_{210}\gamma_{10}\alpha_{0}(1-\pi) + \delta_{211}\gamma_{11}\alpha_{1}\pi}} \\ &= {{\delta_{SF}\Psi_{F} \left[\gamma_{10}\alpha_{0}(1-\pi) + \lambda\gamma_{11}\alpha_{1}\pi\right]}\over{\alpha_{0}(1-\pi)\left[\delta_{SN}\beta_{10}+\delta_{SF}\Psi_{F}\gamma_{10}\right] + \alpha_{1}\pi\lambda \left[\delta_{SN}\beta_{11}+\delta_{SF}\Psi_{F}\gamma_{11}\right]}} \end{array} $$

In the TCC study, the approximate standard error of *V* *E*_*B*_ is:

$$\begin{array}{*{20}l} SE({VE}_{B}) &= SE({OR}_{B}) \approx {OR}_{B}*SE(log({OR}_{B})) \\ &\approx {{p^{B}_{11}\left(1-p^{B}_{10}\right)}\over{p^{B}_{10}\left(1-p^{B}_{11}\right)}} \sqrt{{1\over{N^{B}_{C1}p^{B}_{11}}}+{1\over{N^{B}_{C1}\left(1-p^{B}_{11}\right)}}+{1\over{N^{B}_{C0}}p^{B}_{10}}+{1\over{N^{B}_{C0}\left(1-p^{B}_{10}\right)}}} \end{array} $$

where $N^{b}_{C1}$ is the number of cases and $N^{b}_{C0}$ is the number of controls. The probabilities $\left (p^{B}_{10},p^{B}_{11}\right)$ can be written in terms of the parameters defined earlier:

$${\begin{aligned} p^{B}_{10} &= P(V=1|C_{B}=0, B=1) = {{P(Y=0,V=1)}\over{P(Y=0)}} = {{P(Y=0,V=1)}\over{\sum_{v} P(Y=0,V=v)}}\\ & = {{(1-\gamma_{10}-\beta_{10})\alpha_{0}(1-\pi)+(1-\gamma_{11}-\beta_{11})\alpha_{1}\pi}\over{(1-\pi)[(1-\gamma_{10}-\beta_{10})\alpha_{0} +(1-\gamma_{00}-\beta_{00})(1-\alpha_{0})]+\pi[(1-\gamma_{11}-\beta_{11})\alpha_{1}+(1-\gamma_{01}-\beta_{01})(1-\alpha_{1})]}} \end{aligned}} $$

$${\begin{aligned} p^{B}_{11} &= P(V=1|C_{B}=1, B=1) = {{P(M=1,T=1,V=1)}\over{P(M=1,T=1)}} = {{P(M=1,T=1,V=1)}\over{\sum_{v}P(M=1,T=1,V=v)}}\\ &= {{\delta_{210}\gamma_{10}\alpha_{0}(1-\pi)+\delta_{211}\gamma_{11}\alpha_{1}\pi}\over{\delta_{200}\gamma_{00}(1-\alpha_{0})(1-\pi) + \delta_{201}\gamma_{01}(1-\alpha_{1})\pi + \delta_{210}\gamma_{10}\alpha_{0}(1-\pi) + \delta_{211}\gamma_{11}\alpha_{1}\pi}} \\ &= {{\Psi_{F} \left[\gamma_{10}\alpha_{0}(1-\pi) + \lambda\gamma_{11}\alpha_{1}\pi\right]}\over{\left[\gamma_{00}(1-\alpha_{0})(1-\pi) + \lambda\gamma_{01}(1-\alpha_{1})\pi\right]+\Psi_{F}\left[\gamma_{10}\alpha_{0}(1-\pi) + \lambda\gamma_{11}\alpha_{1}\pi\right]}} \end{aligned}} $$

## Appendix 4

### Unbiasness under random and non-random vaccination

#### Unbiasness under random vaccination

If the vaccination is done at random, then *α*_0_=*α*_1_. The VE estimates can be written as:

$$\begin{array}{*{20}l} {VE}_{A} = 1 - {{\Psi_{F}\left[\gamma_{10}(1-\pi) + \lambda\gamma_{11}\pi\right] \left[\beta_{00}(1-\pi) + \lambda\beta_{01}\pi\right]}\over{\left[\gamma_{00}(1-\pi) + \lambda\gamma_{01}\pi\right]\left[\beta_{10}(1-\pi) + \lambda\beta_{11}\pi\right]}} \end{array} $$

$${VE}_{B} = 1 - {{\Psi_{F}[\gamma_{10}(1-\pi) + \lambda\gamma_{11}\pi][(1-\gamma_{00}-\beta_{00})(1-\pi)+(1-\gamma_{01}-\beta_{01})\pi]}\over{[\gamma_{00}(1-\pi) + \lambda\gamma_{01}\pi][(1-\gamma_{10}-\beta_{10})(1-\pi)+(1-\gamma_{11}-\beta_{11})\pi]}} $$

(1) If *ρ*_*β*_=*Ψ*_*F*_ = 1, and one of the following conditions is satisfied, then *V* *E*_*A*_=*V* *E* *T*_*SI*_.

- *λ*=1;
- *η*_*γ*_=1.

##### *Proof*

Since $\rho _{\beta _{x}} = \Psi _{F}$ = 1, then ${{\beta _{10}}\over {\beta _{00}}} = {{\beta _{11}}\over {\beta _{01}}} = \Psi _{F}$ = 1. We have:

$$\beta_{10} = \beta_{00} \quad \text{and}\quad \beta_{11} = \beta_{01}. $$

So,

$$\begin{array}{*{20}l} {VE}_{A} &= 1 - {{\Psi_{F}[\gamma_{10}(1-\pi) + \lambda\gamma_{11}\pi][\beta_{00}(1-\pi) + \lambda\beta_{01}\pi]}\over{[\gamma_{00}(1-\pi) + \lambda\gamma_{01}\pi][\beta_{00}(1-\pi) + \lambda\beta_{01}\pi]}}\\ &= 1 - {{\gamma_{10}(1-\pi) + \lambda\gamma_{11}\pi}\over{\gamma_{00}(1-\pi) + \lambda\gamma_{01}\pi}} \end{array} $$

If (a) *λ*=1 is satisfied, so

$${VE}_{A} = 1 - {{\gamma_{10}(1-\pi) + \gamma_{11}\pi}\over{\gamma_{00}(1-\pi) + \gamma_{01}\pi}} = {VET}_{SI}. $$

If (b) *η*_*γ*_=1 is satisfied, then *γ*_01_=*γ*_00_ and *γ*_11_=*γ*_10_. Thus,

$$\begin{array}{*{20}l} {VET}_{SI} = 1 - {{\gamma_{10}(1-\pi) + \gamma_{11}\pi}\over{\gamma_{00}(1-\pi) + \gamma_{01}\pi}} = 1 - {{\gamma_{11}}\over{\gamma_{01}}}. \end{array} $$

Hence,

$$\begin{array}{*{20}l} {VE}_{A} &= 1 - {{\gamma_{10}(1-\pi) + \lambda\gamma_{11}\pi}\over{\gamma_{00}(1-\pi) + \lambda\gamma_{01}\pi}} = 1 - {{\gamma_{11}(1-\pi) + \lambda\gamma_{11}\pi}\over{\gamma_{01}(1-\pi) + \lambda\gamma_{01}\pi}} = 1 - {{\gamma_{11}[(1-\pi) + \lambda\pi]}\over{\gamma_{01}[(1-\pi) + \lambda\pi]}} \\ &= 1 - {{\gamma_{11}}\over{\gamma_{01}}} = {VET}_{SI} \quad \quad \text{Q.E.D.} \end{array} $$

(2) If *ρ*_*β*_=1, then *V* *E*_*A*_=*V* *E* *T*_*MAI*_ □

##### *Proof*

Since *ρ*_*β*_=1, then ${{\beta _{10}}\over {\beta _{00}}} = {{\beta _{11}}\over {\beta _{01}}} = 1$.

So,

$$\begin{array}{*{20}l} {VE}_{A} &= 1 - {{\Psi_{F}\left[\gamma_{10}(1-\pi) + \lambda\gamma_{11}\pi\right]\left[\beta_{00}(1-\pi) + \lambda\beta_{01}\pi\right]}\over{\left[\gamma_{00}(1-\pi) + \lambda\gamma_{01}\pi\right]\left[\beta_{00}(1-\pi) + \lambda\beta_{01}\pi\right]}}\\ &= 1 - {{\Psi_{F}\left[\gamma_{10}(1-\pi) + \lambda\gamma_{11}\pi\right]}\over{\gamma_{00}(1-\pi) + \lambda\gamma_{01}\pi}}\\ &= {VET}_{MAI} \quad \quad \text{Q.E.D.} \end{array} $$

(3) If *λ*=1 and 1−*γ*_1*x*_−*β*_1*x*_=*Ψ*_*F*_(1−*γ*_0*x*_−*β*_0*x*_), where *x*=0,1, then *V* *E*_*B*_=*V* *E* *T*_*SI*_ □

##### *Proof*

Since 1−*γ*_1*x*_−*β*_1*x*_=*Ψ*_*F*_(1−*γ*_0*x*_−*β*_0*x*_), where *x*=0,1, and *λ*=1, then

$$\begin{array}{*{20}l} {VE}_{B} &= 1 - {{\Psi_{F}\left[\gamma_{10}(1-\pi) + \lambda\gamma_{11}\pi\right]\left[(1-\gamma_{00}-\beta_{00})(1-\pi)+(1-\gamma_{01}-\beta_{01})\pi\right]}\over{\left[\gamma_{00}(1-\pi) + \lambda\gamma_{01}\pi\right]\left[\Psi_{F}(1-\gamma_{00}-\beta_{00})(1-\pi)+\Psi_{F}(1-\gamma_{01}-\beta_{01})\pi\right]}} \\ &= 1 - {{\gamma_{10}(1-\pi) + \lambda\gamma_{11}\pi}\over{\gamma_{00}(1-\pi) + \lambda\gamma_{01}\pi}}\\ &= 1 - {{\gamma_{10}(1-\pi) + \gamma_{11}\pi}\over{\gamma_{00}(1-\pi) + \gamma_{01}\pi}}\\ &= {VET}_{SI} \quad \quad \text{Q.E.D.} \end{array} $$

(4) If *γ*_1*x*_+*β*_1*x*_=*γ*_0*x*_+*β*_0*x*_, where *x*=0,1, then *V* *E*_*B*_=*V* *E* *T*_*MAI*_ □

##### *Proof*

Since *γ*_1*x*_+*β*_1*x*_=*γ*_0*x*_+*β*_0*x*_, *x*=0,1, then

$$ 1 - \gamma_{1x} - \beta_{1x} = 1 - \gamma_{0x} - \beta_{0x}, \quad \text{where }x = 0, 1 $$

So:

$$\begin{array}{*{20}l} {VE}_{B} &= 1 - {{\Psi_{F}\left[\gamma_{10}(1-\pi) + \lambda\gamma_{11}\pi\right]\left[(1-\gamma_{00}-\beta_{00})(1-\pi)+(1-\gamma_{01}-\beta_{01})\pi\right]}\over{\left[\gamma_{00}(1-\pi) + \lambda\gamma_{01}\pi\right]\left[(1-\gamma_{00}-\beta_{00})(1-\pi)+(1-\gamma_{01}-\beta_{01})\pi\right]}} \\ &= 1 - {{\Psi_{F}\left[\gamma_{10}(1-\pi) + \lambda\gamma_{11}\pi\right]}\over{\gamma_{00}(1-\pi) + \lambda\gamma_{01}\pi}}\\ &= {VET}_{MAI} \quad \quad \text{Q.E.D.} \end{array} $$

□

#### Unbiasness under non-random vaccination

If the vaccination is not done at random, then *α*_0_≠*α*_1_.

(5) If *ρ*_*β*_=*η*_*β*_=*η*_*γ*_=*Ψ*_*F*_=1, then *V* *E*_*A*_=*V* *E* *T*_*SI*_.

##### *Proof*

Since *ρ*_*β*_=*η*_*β*_=*η*_*γ*_=1, then *β*_00_=*β*_10_=*β*_01_=*β*_11_=*Δ* *β*, *γ*_01_=*γ*_00_ and *γ*_11_=*γ*_10_. Thus,

$$ {VET}_{SI} = 1 - {{\gamma_{10}(1-\pi) + \gamma_{11}\pi}\over{\gamma_{00}(1-\pi) + \gamma_{01}\pi}} = 1 - {{\gamma_{11}}\over{\gamma_{01}}}. $$

So,

$$\begin{array}{*{20}l} {VE}_{A} &= 1 - {{\Psi_{F}[\gamma_{10}\alpha_{0}(1-\pi) + \lambda\gamma_{11}\alpha_{1}\pi][\beta_{00}(1-\alpha_{0})(1-\pi) + \lambda\beta_{01}(1-\alpha_{1})\pi]}\over{[\gamma_{00}(1-\alpha_{0})(1-\pi) + \lambda\gamma_{01}(1-\alpha_{1})\pi][\beta_{10}\alpha_{0}(1-\pi) + \lambda\beta_{11}\alpha_{1}\pi]}} \\ &= 1 - {{[\gamma_{11}\alpha_{0}(1-\pi) + \lambda\gamma_{11}\alpha_{1}\pi][(1-\alpha_{0})(1-\pi) + \lambda(1-\alpha_{1})\pi]}\over{[\gamma_{01}(1-\alpha_{0})(1-\pi) + \lambda\gamma_{01}(1-\alpha_{1})\pi][\alpha_{0}(1-\pi) + \lambda\alpha_{1}\pi]}}\\ &= 1 - {{\gamma_{11}[\alpha_{0}(1-\pi) + \lambda\alpha_{1}\pi][(1-\alpha_{0})(1-\pi) + \lambda(1-\alpha_{1})\pi]}\over{\gamma_{01}[(1-\alpha_{0})(1-\pi) + \lambda(1-\alpha_{1})\pi][\alpha_{0}(1-\pi) + \lambda\alpha_{1}\pi]}} \\ &= 1 - {{\gamma_{11}}\over{\gamma_{01}}} = {VET}_{SI} \quad \quad \text{Q.E.D.} \end{array} $$

(6) If *ρ*_*β*_=1 and *η*_*β*_=*η*_*γ*_, then *V* *E*_*A*_=*V* *E* *T*_*MAI*_. □

##### *Proof*

Since *η*_*β*_=*η*_*γ*_, so ${{\beta _{01}}\over {\beta _{00}}} = {{\beta _{11}}\over {\beta _{10}}} = {{\gamma _{01}}\over {\gamma _{00}}} = {{\gamma _{11}}\over {\gamma _{10}}}$. Then we have: ${\gamma _{11}\over {\beta _{11}}} = {\gamma _{10}\over {\beta _{10}}} \stackrel {\Delta }{=} a, {\gamma _{00}\over {\beta _{00}}} = {\gamma _{01}\over {\beta _{01}}} \stackrel {\Delta }{=} b$, and ${{\beta _{10}}\over {\beta _{00}}} = {{\beta _{11}}\over {\beta _{01}}} = 1$. Then:

$$\begin{array}{*{20}l} {VE}_{A} &= 1 - {{\Psi_{F}\left[\gamma_{10}\alpha_{0}(1-\pi) + \lambda\gamma_{11}\alpha_{1}\pi\right]\left[\beta_{00}(1-\alpha_{0})(1-\pi) + \lambda\beta_{01}(1-\alpha_{1})\pi\right]}\over{\left[\gamma_{00}(1-\alpha_{0})(1-\pi) + \lambda\gamma_{01}(1-\alpha_{1})\pi\right]\left[\beta_{10}\alpha_{0}(1-\pi) + \lambda\beta_{11}\alpha_{1}\pi\right]}}\\ &= 1 - {{\Psi_{F}\left[a\beta_{10}\alpha_{0}(1-\pi) + a\lambda\beta_{11}\alpha_{1}\pi\right]\left[\beta_{00}(1-\alpha_{0})(1-\pi) + \lambda\beta_{01}(1-\alpha_{1})\pi\right]}\over{\left[b\beta_{00}(1-\alpha_{0})(1-\pi) + b\lambda\beta_{01}(1-\alpha_{1})\pi\right]\left[\beta_{10}\alpha_{0}(1-\pi) + \lambda\beta_{11}\alpha_{1}\pi\right]}} \\ &= 1 - \Psi_{F} \cdot {a\over b} \end{array} $$

and,

$$\begin{array}{*{20}l} {VET}_{MAI} &= 1 - {{\Psi_{F} \left[\gamma_{10}(1-\pi) + \lambda\gamma_{11}\pi\right]}\over{\gamma_{00}(1-\pi) + \lambda\gamma_{01}\pi}} = 1 - {{\Psi_{F}\left[a\beta_{10}(1-\pi) + a\lambda\beta_{11}\pi\right]}\over{b\beta_{00}(1-\pi) + b\lambda\beta_{01}\pi}}\\ &= 1 - \Psi_{F} \cdot {a\over b} \end{array} $$

So, *V* *E*_*A*_=*V* *E* *T*_*MAI*_. □

## Abbreviations

ARI: Acute respiratory illness
FARI: Influenza ARI
MAI: Medically-attended influenza
NFARI: Non-influenza ARI
OR: Odds ratio
SE: Standard error
SI: Symptomatic influenza
TCC: Traditional case-control
TN: Test-negative
VE: Vaccine effectiveness
